# On Morbid Excess of Vascular Distention

**Published:** 1817-11

**Authors:** 


*For the London Medical and Physical Journal.*

*On Morbid Excess of Vascular Distention;* by Dr. KINGLAKE.

EXCESSIVE vascular distention may be justly regarded as a fertile source of diseases in the animal economy. A given extent of distended vessel is essential to life and health; and it is only considerable deviations from the natural standard of this state that either dispose to, or actually constitute, disease. The principle of distention is the most general and the most indispensable to vital action: it is by its agency that the native powers of life are excited, and continued in action. The healthful state of vascular distention is that which is natural: neither excess nor deficiency in this important condition of life can occur without inducing more or less of disease. If the distention be beyond the natural limits of vascular action, it will be accompanied

---

with with immoderate excitement; if it should be within the healthful bounds, it will be denoted by deficient excitement: thus the two opposite states of excessive and insufficient exertion of vital power essentially depend on the existing degree of vascular distention. It is an inherent property of the arterial system to be stimulated by the distending impulse of its circulating fluids. In obedience to this law, the heart is acted on; and the effect of its contractile exertions is, in some measure, extended throughout the whole sanguiferous system. The blood also appears to possess a stimulating quality, perhaps derived from the oxygenous admixture imparted to it in its passage through the lungs; but this would be insufficient, unaided by the influence produced on the contractility of the arterial structure by the mechanic stimulus of distention. The contractile power of the arterial system is incessantly exerted so as to adapt its tubular cavities to the quantity of their contained fluid: hence the blood-vessels may be regarded as always full, and, of course, always more or less excited by the stimulus of distention. When the area of the blood-vessels is much narrowed by progressive contraction on their diminished contents, a great expenditure of vital power has been incurred, and the force of distention is greatly lessened. In such circumstances, therefore, a sense of debility and exhaustion is induced, and evinced by diminished energy in the arterial action. Excepting in instances of accidental and sudden escape of the sanguineous fluids, the contents of the vascular system are uniformly sufficient to exert a strong distending force on the transmitting vessels: when this quantity is hurried on more rapidly than is consistent with health, every portion of the arterial system becomes inordinately distended, and diseases of an acutely inflammatory description are apt to be produced; so also, if the vascular energies have been diminished, and the circulation retarded, an undue degree of vascular distention may arise, inducing partially increased actions in certain portions of the system of the chronic and congestive kind of inflammatory disease. Unbroken vigour and strength are not requisite to constitute inflammatory disease. This morbid state will often arise from the stimulus of distention under circumstances of both general and local debility. The diseased state consists of inordinate excitement; and, until the healthful condition be restored, the increased action will go on, and will only end with the life or disorganization of the affected part.

Acute diseases of all kinds, however they may have originated, or by whatever name distinguished, are generally connected with a morbidly distended state of vessel, requiring depletion depletion for relief, and for preventing the organic and general mischief which such morbid violence is always likely to produce. It is not often known precisely in what part of the system morbid excitement commences, nor is the existence of disease suspected or noticed until its baneful influence has been extended to the action of the heart and arteries,—until the throbbing rapidity of their action announces its insufferable severity. The constant endeavour should be to repress and subdue the diseased excitement as soon as possible, and thus to anticipate the healthful restoration which the morbid conflict itself is, perhaps, designed to accomplish. Noxious and friendly powers to health prove morbidly stimulant: the excitement induced tends to expend the vital power that gives effect to the morbid cause, and thus to regain the healthful state. Excessive vascular distention, whether attended with increased or diminished arterial action, is a state of disease requiring the compressive and counteracting aid of depletion. By diminishing the vascular contents, the distended vessels are enabled to resume their natural dimensions, and to overcome the straining violence to which such an unnatural state of dilatation had subjected them. What obtains in this way by the natural contractility of the vessel, is afforded in various local ailments by external pressure and confinement. Ulcerous inflammation, tumefactions, tendinous and ligamentous enlargements, and even cancerous diseases, are said to have been benefitted by pressure. In these cases the distended vessels have been compressed, and brought within their natural and healthy sphere of action, when pain, extravasation, and morbid accretions have been relieved and cured. The influence of external pressure in amending inflamed, enlarged, indurated, and disorganised parts near the surface, is precisely similar to what takes place in enabling the morbidly-distended vessels, in internal affection, to resume the natural scope of their healthy action by diminishing their contents; in both instances the condition approaching to tearing asunder or lacerating the continuity of organic structure is removed, and the easy and calm state of healthy action substituted in its stead.

The principle of vascular distention has not been sufficiently regarded by pathologists in their estimates and speculations on morbid action. In referring every thing to the abstract energy of vital power, the mechanism of its action has been too much overlooked. A revered sufficiency has been assigned to its general efficacy, whilst its various evolutions and modes of action have been considered as too mysterious mysterious for human comprehension. The effect of this neglect in medical practice has been to consecrate the doctrines of tone and debility, as affording a satisfactory explanation of all the morbid phenomena that are liable to occur. Had the diversified circumstanccs of diseased action been more referred to the various states of comparative repletion and inanition, a more direct and efficient mode of treatment would have been proposed and practised. The diseases originating in undue vascular distention, however induced, or in whatever degree existing, would then be directly combated by an appropriate remedy, whilst those which arose from a deficient state of that healthful condition of life would be aided by means suited to afford immediate relief. Numerous are the acute as well as chronic forms of disease that have their source in an unhealthful state of vascular distention. The more violent or acute instances of this kind are readily noticeable; their symptoms are not ambiguous, but distinctly exhibit their true nature. Those of a chronic description are less evident: they indeed are often so enveloped in general debility, and in various indications of visceral disease, as greatly to obscure the precise character and extent of the affection. As in all other cases of disease, an ample scope is here afforded for rational contemplation; and the experienced and discerning practitioner will have but little difficulty in determining, by a mature consideration of the attending circumstances, in what the disease mainly consists, and how its cure should be attempted. On the one hand, abstinence from alcoholic stimulants of every kind, however disguised or named, vascular and alvine discharges to a suitable extent, sufficient dilution with aqueous fluids to determine to the surface, and an unoppressive nutritive diet, will conjointly overcome the various distressing effects of vascular distention; whilst, on the other, restraining inordinate evacuations, a nutritious diet, moderate personal exertion, and, where more active excitement may be indicated, a limited use of fermented liquors, would speedily restore to the healthy standard such morbid states of relative inaction and emptiness of vessel as may occur in diseases of weakness and depletion.

*Taunton; August 5,* 1817.

